# Effects of increased temperature on plant communities depend on landscape location and precipitation

**DOI:** 10.1002/ece3.3995

**Published:** 2018-05-08

**Authors:** Jane Cowles, Bazartseren Boldgiv, Pierre Liancourt, Peter S. Petraitis, Brenda B. Casper

**Affiliations:** ^1^ Department of Biology University of Pennsylvania Philadelphia PA USA; ^2^ Department of Ecology, Evolution & Behavior University of Minnesota Twin Cities Saint Paul MN USA; ^3^ Department of Biology National University of Mongolia Ulaanbaatar Mongolia; ^4^ Institute of Botany Academy of Sciences of the Czech Republic Třeboň Czech Republic

**Keywords:** biodiversity, context dependency, global change experiment, open‐top chambers, precipitation, primary productivity

## Abstract

Global climate change is affecting and will continue to affect ecosystems worldwide. Specifically, temperature and precipitation are both expected to shift globally, and their separate and interactive effects will likely affect ecosystems differentially depending on current temperature, precipitation regimes, and other biotic and environmental factors. It is not currently understood how the effects of increasing temperature on plant communities may depend on either precipitation or where communities lie on soil moisture gradients. Such knowledge would play a crucial role in increasing our predictive ability for future effects of climate change in different systems. To this end, we conducted a multi‐factor global change experiment at two locations, differing in temperature, moisture, aspect, and plant community composition, on the same slope in the northern Mongolian steppe. The natural differences in temperature and moisture between locations served as a point of comparison for the experimental manipulations of temperature and precipitation. We conducted two separate experiments, one examining the effect of climate manipulation via open‐top chambers (OTCs) across the two different slope locations, the other a factorial OTC by watering experiment at one of the two locations. By combining these experiments, we were able to assess how OTCs impact plant productivity and diversity across a natural and manipulated range of soil moisture. We found that warming effects were context dependent, with the greatest negative impacts of warming on diversity in the warmer, drier upper slope location and in the unwatered plots. Our study is an important step in understanding how global change will affect ecosystems across multiple scales and locations.

## 1 INTRODUCTION

Major climatic shifts in temperature and precipitation will continue to affect the ecology of natural systems worldwide. Temperatures are expected to increase globally with larger changes in higher latitudes (IPCC 2013), while changes in precipitation are expected to be less consistent and predictable (IPCC 2013). Indeed, some regions have already documented increases in precipitation, while others have shown marked decreases and still others are experiencing changes in the frequency and intensity of precipitation events (Goulden et al., 2016; Vandandorj, Munkhjargal, Boldgiv, & Gantsetseg, 2017). In particular, because temperature and precipitation are not expected to change in parallel, discerning the ecological consequences of climate change will require understanding the consequences of elevated temperature at different levels of soil moisture or precipitation.

Climate change experiments frequently manipulate either temperature or precipitation but not both despite findings that multiple global change factors can interactively affect the same ecosystem processes (Blumenthal, Kray, Ortmans, Ziska, & Pendall, 2016; Cowles, Wragg, Wright, Powers, & Tilman, 2016; Reich et al., 2001; Sherry et al., 2008; Yang, Li, et al., 2011; Yang, Wu et al., 2011). In one notable exception, Zhu, Chiariello, Tobeck, Fukami, and Field (2017) manipulate four important global change factors over 17 years and found complex, interactive, and nonlinear effects between the factors. Moreover, soil moisture and temperature are coupled such that studies manipulating one often manipulate the other. For example, warming devices can have drying effects due to rainfall interception (Carlyle, Fraser, & Turkington, 2011) and, on a more basic level, due to enhanced evaporation associated with rising temperatures (Vicente‐Serrano, Beguería, & López‐Moreno, 2010). As a result, we currently do not fully understand the interactive impacts of warming and precipitation changes on ecosystems nor do we understand how the consequences of increased temperature may differ depending on soil moisture status, due to this high level of coupling.

Comparisons of disparate manipulative climate experiments across regional and global temperature gradients or across precipitation gradients provide evidence that the impact of changes in temperature on productivity depends on soil moisture (Wu, Dijkstra, Koch, Penuelas, & Hungate, 2011; Elmendorf et al., 2011, 2012). Indeed, the productivity response to experimental warming varies with soil moisture, but not in a consistent manner (Elmendorf et al., 2011, 2012; Wu et al., 2011). Other ecosystem factors, such as species composition, and environmental factors like soil type and infiltration rates (Dieleman et al., 2012; Way & Oren, 2010) are likely also important and potentially explain the inconsistent relationship between soil moisture and the response to increased temperature. Furthermore, experimental warming tends to increase productivity more strongly near the poles (Rustad, Campbell, Marion, Norby, & Mitchell, 2001), indicating another context dependency of warming effects. Different experiments can produce different results for a multitude of reasons, including not only design and implementation, but also site‐level differences. The context dependency of warming effects on productivity is a question that clearly requires further examination.

Like the productivity response, the response of diversity and community composition to warming is also not consistent across variation in ambient soil moisture (Elmendorf et al., 2011). Generally, experimental warming often leads to reductions in diversity (Klein, Harte, & Zhao, 2004; Gedan & Bertness, 2009; Prieto, Penuelas, Lloret, Llorens, & Estiarte, 2009; but see Zavaleta et al., 2003; Harmens et al., 2004; Yang, Wu et al., 2011) and changes in community structure (Cowles et al., 2016). Experimentally increased precipitation, likewise, can have either a positive or negative impact on plant diversity (Báez, Collins, Pockman, Johnson, & Small, 2012; Xu et al., 2012). In one manipulative study, the diversity response depended on the dominant plant taxa of the system (Báez et al., 2012). Thus, examining how plant communities respond to both warming and changes in precipitation in a factorial experiment and in different communities is vital for a more complete understanding of the impacts of global changes.

Here, we report the results of a study in the steppe of northern Mongolia in which experimental temperature manipulation is applied at two slope locations and crossed with experimental water addition at the drier location. The two sites differ in elevation, aspect, and plant community structure. Our goal was to understand how predicted changes in temperature will interact with predicted changes in precipitation (Bayasgalan et al., 2009) and natural variation in soil moisture to affect ecosystems. Using both simulated precipitation shifts and multiple locations, we increase our ability to understand the context dependency of temperature impacts. We examine how warming and added precipitation affect soil temperature and moisture and how these soil factors, in turn, affect plant productivity and diversity. Furthermore, as the treatments effectively make the two locations more abiotically similar to one another, we can assess the utility of space‐for‐time substitutions in the systems: whether moving up the slope is akin to future warming scenarios.

We focus on two plant community metrics, biomass productivity and plant species diversity, because of the importance of primary productivity in supporting higher trophic levels (Lindeman, 1942) and providing storage of atmospheric carbon and other ecosystem services (Millenial Ecosystem Assessment, 2005) and because of the role of plant diversity in contributing to a healthy, stable ecosystem (Loreau & de Mazancourt, 2013; Tilman, Isbell, & Cowles, 2014; Tilman, Reich, & Knops, 2006). Climate was manipulated using open‐top chambers (OTCs), which elevate temperature but also have a drying effect (Liancourt et al., 2012; Marion et al., 1997). As such, we utilized the two locations naturally differing in soil moisture and temperature to examine the context dependency of warming. Further, at the naturally drier locations, we crossed the warming treatment with water addition to simulate increased precipitation. We expected that warming and drying from the OTC (Liancourt et al., 2012) would negatively impact plant productivity and diversity, and especially so in the warmer, drier location, where plants might be operating closer to their thermal optima (Wertin, Reed, & Belnap, 2015), and be more water limited. As such, we would expect the drying effects of the warming treatment to be more consequential than the temperature effects (Dieleman et al., 2012). We further hypothesized that additional precipitation could moderate much of the negative impact of the OTC.

## 2 METHODS

### 2.1 Study site and experimental design

The experiment was conducted from 2009 to 2012 in two locations on a south‐facing slope in the Dalbay River valley in the Mongolian steppe (51°01.405′N, 100°45.600′E). Regionally, the average annual air temperature is −4.5°C, ranging from an average monthly temperature of −21°C in January and 12°C in July (Nandintsetseg, Greene, & Goulden, 2007). The growing season extends from mid‐June to mid‐August. Average annual precipitation, measured over a recent 40 year period, is 265 mm (Namkhaijanstan, 2006).

The locations were separated by 300 m and differed in elevation, aspect, and microclimate. The upper slope location, which was ~40% drier than the lower slope (Liancourt et al., 2012), was at 1,800 m a.s.l. and on a ~20° incline, while the lower slope location was at 1,670 m a.s.l. and at a flat or gentle incline. The growing season night time temperature was around 3°C colder on the lower slope location (5.3°C vs. 8.4°C at the upper slope (Liancourt et al., 2013), while the daytime air temperature was approximately equivalent (~15°C). The composition of the vegetation also differed somewhat. The lower slope was dominated by the sedge *Carex pediformis*, while the abundance of species on the upper slope is more even; *C. pediformis* and forbs *Oxytropis strobilacea* and *Potentilla acaulis* had, on average, roughly the same abundances in a census conducted in the first year of the experiment.

On the upper, drier slope, the OTC treatment was crossed with a supplemental watering treatment, which resulted in four treatment combinations: (OTC treatment & watered; OTC treatment & unwatered; control (no OTC) & watered; control (no OTC) & unwatered). Spatially, one replicate of each of the four treatment combinations was placed within a separate 9 m × 9 m fenced area, which we treated statistically as a block. There were seven replicate blocks total on the upper slope separated by at least 30 m. There were eight replicates, similarly spaced blocks on the lower slope. A grazing treatment on the lower slope was not included in this study (i.e. all plots included in this study were fenced); the grazing treatment plots are described elsewhere (Spence et al. 2014).

Here, we took advantage of the two slope locations to conduct, through our statistical analyses, two separate experiments. In the first experiment, we examined the factorial combinations of climate manipulation (OTC vs. control) and slope location (upper vs. lower) in order to determine if the effects of climate manipulation differed within a landscape. We were unable to replicate slopes, which would have required travel to different valleys and was logistically impossible. Thus, the elevation treatment is pseudoreplicated and ecological inferences about the effect of slope are potentially confounded with other unknown sources of environmental variation. Note, however, the replicate blocks spread out over approximately 240–280 m on the upper and on the lower slope and our design should sample effectively the differences between the two slope locations. The second experiment examined the effects of the climate manipulation (OTC vs. not) crossed with the precipitation treatments (watered vs. not) on the upper slope only.

The OTCs, as passive warming devices (see Marion et al., 1997), were constructed using Sun‐Lite^®^ HP fiberglass glazing mounted on a clear Lexan frame. They were hexagonal in shape, 40 cm tall, 1.5 m wide at the bottom, and 1.0 m wide at the opening due to the inward sloping sides of the chambers. Control plots were identical in footprint size to the OTCs.

On average, OTCs elevated air temperature by 1.5°C in the day and decreased temperature by −0.2°C at night (Liancourt et al., 2013). Weather conditions can affect the strength of the OTC effect (Bokhorst et al., 2013), and we saw much variation around the mean throughout the growing season and times of day (Figure S1). Still, the mean effects provide an integrative look at the effects of warming over the course of the growing season. In addition to the warming effects, the chambers reduced volumetric soil moisture by 30%, largely due to rain interception rather than greater evaporation (see Liancourt et al., 2012). While no experimental temperature manipulation in the field can accurately recreate predicted climate warming effects (Amthor, Hanson, Norby, & Wullschleger, 2010; Aronson & McNulty, 2009; Rich et al., 2015), OTCs are the best and most feasible option available for any remote location without access to electricity (Aronson & McNulty, 2009). Each year, the OTCs were set up after the last snow but prior to onset of most new growth (early June) and were kept in place until late July—mid‐August. The only early termination (i.e. late July) was 2012 when the plants were harvested and the experiment terminated.

Supplemental precipitation on the upper slope simulated an additional 4.5 mm weekly rainfall event above ambient. Treatment plots were watered once weekly in the evening using water from the nearby Dalbay River. This treatment was applied for 7 weeks in 2009, 10 weeks in 2010, 9 weeks in 2011, and 7 weeks in 2012. Note that we could not predict, in advance, by what percentage supplemental watering would increase the yearly amount. The supplemental precipitation increased the seasonal precipitation by 15.7%, 25.3%, 29.6%, and 30.2% for those years, respectively.

### 2.2 Measured variables

Because we were particularly interested in the combined effects of multiple years of treatments, we examined productivity and plant species diversity per plot in terms of percent cover and biomass in the final year of the experiment, 2012. Percent cover by species was ascertained in mid‐July using a 50 cm × 100 cm gridded (10 cm × 10 cm) quadrat centered in each experimental plot, with the short side of the rectangle parallel with the northern side of the hexagonal plot. Percent cover for each species was estimated to the nearest 10%, and values for a given species were averaged across all grid cells to obtain the average percent cover per species per plot. Total cover for each plot is the summation of percent cover for all species and could thus be >100% due to species overlap. In late July of 2012, we harvested biomass in the same 50 cm × 100 cm areas where we conducted the vegetation cover surveys. We clipped plants at ground level, sorted to species, and air‐dried the biomass in the field. Upon returning to the laboratory, we oven dried the samples for 36 hr at 80°C and weighed the biomass thereafter. We estimated total aboveground biomass by adding the biomass of all species and multiplying by two to achieve units of grams per square meter.

We calculated Shannon diversity (H′) based on vegetative cover per species (cover diversity) and based on aboveground biomass per species (biomass diversity). We then back transformed using an exponential function so the units of diversity are expressed as species equivalents (Hill, 1973). In each case, we refer to our final diversity metric (*e* ^H′^) simply as “diversity.”

We measured three environmental variables within the experimental plots and examined them as possible predictor values for plant productivity and community diversity. These were available nitrogen, soil temperature, and soil moisture. To measure plant available nitrogen, we used plant root simulator (PRS)^™^ probes (Western Ag Innovations Inc., Saskatoon, SK, Canada; https://www.westernag.ca/innov). Two anion and two cation probes were inserted at the soil surface (0–6 cm) along a long edge of the vegetative sampling area in each plot and remained in place for 21 days in the middle of the growing season. After retrieval, probes were brushed in the field to remove soil and later washed in the laboratory with deionized water. Probes were analyzed by Western Ag Innovations, yielding concentrations (in μg N per 10 cm^2^ ion exchange surface per day) of soil available inorganic nitrogen (the sum of ${\text{NO}}_{3}^{-}$ and ${\text{NO}}_{4}^{+}$).

We measured soil temperature (°C) and volumetric soil moisture at 0–6 cm depth 16 times throughout the 2012 season using a portable probe (WET‐2 sensor, Delta‐T Devices Ltd., Cambridge, UK). Ten of these days were within an 11‐day span in June (June 20–30), and six were in the 7‐day span between July 12 and 18. For each day, data were collected at three points along a transect within each plot, which were averaged to get one value per plot. Analyses of the temporal dynamics of soil moisture in OTC plots and controls on both slope locations for the first 2 years of the experiment are presented in (Liancourt et al., 2012).

### 2.3 Statistical analyses

All statistical analyses were conducted in R 3.3.0 (http://www.r-project.org). In all cases, linear mixed effect models were applied, utilizing the lme function from the package *nlme* (Pinheiro, Bates, DebRoy, & Sarkar, 2017), with block (a cluster of one replicate plot of each treatment and control) as a random effect. We examined how our treatments and their interaction affected available nitrogen, soil moisture, soil temperature, plot level biomass (biomass), plot level % cover (cover), diversity (*e* ^H′^) calculated from plant biomass measurements (biomass diversity), and diversity (*e* ^H′^) calculated from plant % cover measurements (cover diversity). Response variables were assessed for normality and transformed if necessary. Thus, only cover was log_e_ transformed; all other responses were not transformed. We ran a separate analysis for each response variable within each experiment—OTC treatment (OTC) at the two slope locations (Slope) and OTC treatment (OTC) crossed with supplemental precipitation (Water) at the upper slope location. The upper slope data used for the OTC × slope location are the same as the no water treatment for the OTC × water experiment.

In addition to the above‐described models assessing the impacts of the treatments and their interactions on all measured variables (referred to here as ANOVA due to the lack of environmental covariates), we ran ANCOVA models for the biotic response variables. These mixed effects models included the primary axis from a PCA including soil moisture and soil temperature as covariate in addition to the treatments and their interactions. Because temperature and moisture are highly correlated, we utilized the primary axis (PC1) from a PCA analysis to avoid including multiple correlated variables. Nitrogen was not included as our method of detection was heavily driven by changes in moisture. The PCA axis explained 97.6% and 95.4% of the variance in the PCA for the OTC × Slope and OTC × Water experiments, respectively. For both of the PCAs, temperature and moisture loadings were in opposite directions along PC1. For OTC × Slope, temperature was negatively correlated with PC1 while moisture was positively correlated with PC1. For the OTC × Water experiment, this was reversed. As such, the PC1 variable for OTC × Water was multiplied by −1 to allow for the consistent interpretation of this variable: Positive levels of PC1 are correlated with cool and wet. We included this axis as a covariate to assess whether accounting for covariation in abiotic factors changes the pattern of significance of the treatments and thus alter our inferences about the underlying mechanisms of the treatment themselves on the biotic response variables. For example, a change from a significant effect of Slope in the ANOVA to a nonsignificant effect in the ANCOVA would suggest slope‐specific differences in soil temperature or moisture were driving the significant slope differences seen in the ANOVA. Conversely a nonsignificant effect in the ANOVA but a significant effect in the ANCOVA would suggest that the abiotic effects included in the covariate confounded and obscured additional slope‐specific effects unrelated to temperature and moisture.

Pearson's correlation coefficients were calculated for both experiments to examine the relative strength of correlations between the biotic variables and the abiotic variables of interest (temperature and moisture). Because temperature and moisture are highly correlated themselves, these relationships are solely discussed as correlations.

## 3 RESULTS

### 3.1 OTC × slope experiment

**Figure 1:**
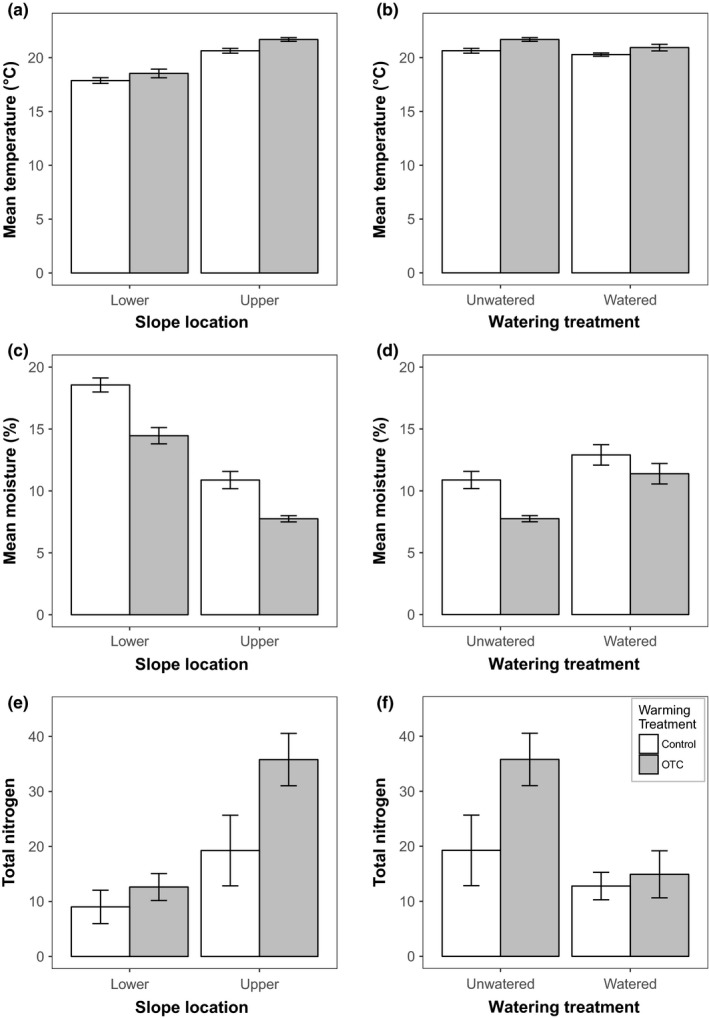
Effects of experimental treatments on abiotic properties. The left panel illustrates the effects of open‐top chamber (OTC) treatment (OTC vs. Control) and slope location (lower vs. upper slope) on (a) average soil temperature, (c) average soil moisture, and (e) total plant available nitrogen (${\text{NO}}_{3}^{-}+{\text{NO}}_{4}^{+}$) across the 2012 growing season. The right panel shows the effect of climate manipulation (OTC vs. control) and precipitation (control vs. added water) on (b) average soil temperature, (d) average soil moisture, and (f) total plant available nitrogen (${\text{NO}}_{3}^{-}+{\text{NO}}_{4}^{+}$). Error bars are standard error of the mean. Note that the upper slope location in the left graphs (OTC × slope experiment) is the same plots as the unwatered treatment in the right panel (OTC × water experiment). They are aligned for comparison, but due to the experiment design are analyzed as distinct models

Our measured environmental covariates differed between the slope locations and were affected by the OTC treatments. The upper slope location exhibited elevated soil temperature (*p* < .0001, Figure 1a), lower soil moisture (*p* < .0001, Figure 1c), and greater soil available nitrogen (*p* < .001, Figure 1e) relative to the lower slope location. The increased soil available nitrogen was largely driven by a difference in nitrate (*p* < .001), as no effect of slope location on ammonium was detected (*p* = .707). OTCs significantly increased soil temperature (*p* < .0001, Figure 1a), decreased soil moisture (*p* < .0001, Figure 1c), and increased soil available nitrogen (*p* < .01, Figure 1e). The effect of OTCs on these environmental variables was not significantly different at the two locations (i.e. no OTC × slope interaction).

**Figure 2:**
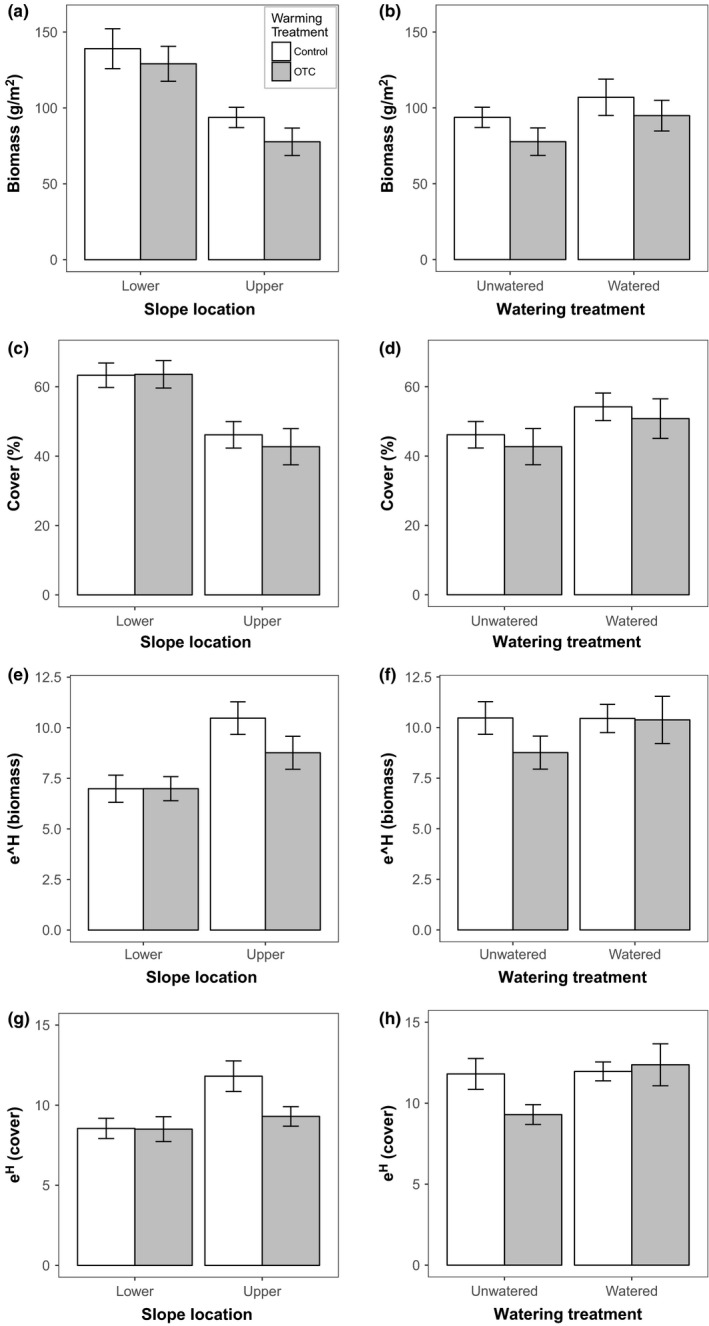
The effect of open‐top chambers (OTCs) (OTC vs. Control) and slope location (lower vs. upper slope; left panel) and OTCs and watering treatment (right panel) on productivity based on biomass (a,b) and percent cover (c,d) and diversity (*e* ^H′^) calculated based on biomass data (e,f) and total percent cover data (g,h). Means shown are unadjusted averages for each treatment. Error bars are standard error of the mean. Note that the Upper slope location in the left graphs (OTC × slope experiment) is the same plots as the unwatered treatment in the right panel (OTC × water experiment). They are aligned for comparison, but due to the experiment design are analyzed as distinct models

Open‐top chamber treatment affected community biomass and diversity. OTCs marginally decreased productivity when measured as biomass but not when measured in terms of percent cover (Table 1, Figure 2a,c). There were, however, significant main effects of both OTCs and slope location on cover diversity and a significant interactive effect between the two. This interaction reflects a negative effect of OTCs on diversity but only at the warmer, drier upper slope (Figure 2g). Plant communities on the upper slope were significantly less productive and more diverse than those on the lower slope, both in terms of biomass and percent cover (Figure 2). Furthermore, when PC1 (including information about moisture and temperature) was included in the model, the treatment effects were obfuscated in multiple cases, indicating that some of the variability leading to the impact of treatments on the response variables can be explained by the temperature and moisture changes (Table 1).

**Table 1:** Model results for the OTC × slope experiment, where OTC and control plots were set up at two locations on the slope

Variable	Source	ANOVA	ANCOVA	Sign of effect
Biomass	OTC	0.0792	0.3171	NA
	Slope location	**0.0003**	0.0879	NA
	OTC: slope location	0.6752	0.7020	NA
	PC1	NA	0.9032	(−)
		AIC: 265.1	AIC: 259.5	
Cover	OTC	0.1904	0.5494	NA
	Slope location	**0.0003**	**0.0441**	NA
	OTC: slope location	0.1482	0.1477	NA
	PC1	NA	0.7949	(−)
		AIC: −1.9	AIC: 2.7	
Biomass diversity	OTC	0.2304	0.3609	NA
	Slope location	**0.0006**	0.3098	NA
	OTC: slope location	0.1977	0.2117	NA
	PC1	NA	0.9334	(−)
		AIC: 128.7	AIC: 128.4	
Cover diversity	OTC	**0.0378**	0.3791	NA
	Slope location	**0.0232**	0.2737	NA
	OTC: slope location	**0.0329**	**0.0264**	NA
	PC1	NA	0.5482	(+)
		AIC: 128.9	AIC: 128.2	

Values are *p*‐values of model coefficients (bolded if *p* < .05) and the sign of effect indicates whether the variable had a positive or negative impact on the response. AIC scores for each model are reported.

Across both slope locations and the OTC treatment, we found a large and significant correlation between temperature and moisture, as well as between both temperature and moisture and our plant response variables. For all plant response variables, the correlation between the plant response variable and temperature was larger than the correlation between the plant response variable and moisture (Table 3).

### 3.2 OTC × water experiment (upper slope only)

Open‐top chambers and supplemental precipitation (Water) both impacted soil temperature, moisture, and available nitrogen. Our OTC treatment increased soil temperature (*p* < .0001, Figure 1b) and decreased soil moisture (*p* < .0001, Figure 1d), just as seen in the analyses that included both slope locations. Watering increased soil moisture as expected (*p* < .0001, Figure 1d) and decreased soil temperature (*p* < .001, Figure 1b). There was an interactive effect of OTC and water on soil moisture, such that the positive effect of watering was greater inside the OTCs (*p* < .05, Figure 1d). OTCs increased total available nitrogen (*p* < .05, Figure 1f) and watering decreased total available nitrogen (*p* < .01, Figure 1f). The positive effect of OTCs on available nitrogen was reduced by watering (OTC × water interaction: *p* < .05, Figure 1f). These effects on available nitrogen were again driven by shifts in nitrate, as no significant effects of treatments on ammonium were observed (not shown).

On the upper slope, OTCs marginally decreased plant biomass (*p* = .08, Figure 2b) and watering marginally increased biomass (*p* = .06, Figure 2b). Watering significantly increased plant cover (*p* < .05, Figure 2d), but OTCs had no significant effect on cover. We found a marginally significant negative effect of watering treatment on cover diversity (*p* = .06, Figure 2h) but no significant effect of OTC treatment on cover diversity. There was a marginally significant interactive effect of OTC and watering on cover diversity (*p* = .09, Figure 2h) reflecting a weakening of the OTC effect on cover diversity when water was added. When including abiotic covariates (as PC1) in the model, we found significant impacts of both treatments on plant biomass and of watering on plant cover (Table 2).

**Table 2:** Model results for the OTC × water experiment, where plots with or without OTCs received supplemental precipitation or not

Variable	Source	ANOVA	ANCOVA	Sign of effect
Biomass	OTC	0.0818	**0.0008**	NA
	Watering	0.0599	**0.0003**	NA
	OTC: watering	0.8060	0.2862	NA
	PC1	NA	**0.0005**	(−)
		AIC: 240.9	AIC: 229.3	
Cover	OTC	0.2799	0.0690	NA
	Watering	**0.0323**	**0.0088**	NA
	OTC: watering	0.9254	0.5918	NA
	PC1	NA	0.0906	(−)
		AIC: 17.6	AIC: 20.1	
Biomass diversity	OTC	0.3137	0.3363	NA
	Watering	0.3685	0.3962	NA
	OTC: watering	0.3530	0.3159	NA
	PC1	NA	0.6168	(−)
		AIC: 128.9	AIC: 128.9	
Cover diversity	OTC	0.2258	0.6771	NA
	Watering	0.0629	0.4454	NA
	OTC: watering	0.0928	0.1303	NA
	PC1	NA	0.7089	(+)
		AIC: 129.7	AIC: 129.7	

Values are *p*‐values of model coefficients (bolded if *p* < .05) and the sign of effect indicates whether the variable had a positive or negative impact on the response. AIC scores for each model are reported.

On the upper slope, we observed much larger correlations between plant response variables and temperature than between the plant response variables and moisture (Table 3). Moisture had a larger correlation with the plant diversity variables than with the biomass variables (Table 3). Interestingly, while we found a positive effect of temperature on diversity and a negative effect of moisture on diversity in the cross‐slope experiment, we found the reverse to be true in the water addition experiment on the upper slope only (Table 3).

**Table 3:** Correlations between soil measurements and plant response variables across all experimental treatments in each experiment

	Soil temperature	Soil moisture
OTC × slope experiment		
Total plant cover (%)	−.66	.52
Total plant biomass (g/m^2^)	−.80	.61
Cover diversity (*e* ^H′^)	.33	−.24
Biomass diversity (*e* ^H′^)	.49	−.41
OTC × water experiment		
Total plant cover (%)	−.37	.02
Total plant biomass (g/m^2^)	−.47	−.08
Cover diversity (*e* ^H′^)	−.52	.33
Biomass diversity (*e* ^H′^)	−.33	.14

Values shown are Pearson's *r*.

## 4 DISCUSSION

Our study showed that open‐top chambers tended to reduce productivity and diversity, while increased precipitation had a positive effect on these variables. Importantly, the response to open‐top chambers was context dependent: The warmer, drier upper slope was more vulnerable to chamber‐induced diversity loss than the lower slope, and supplemental precipitation reduces that vulnerability. Our work highlights the utility of using both natural and manipulated variability in soil moisture in an experimental framework. We were able to analyze, at a variety of moisture levels, how temperature affects plant communities by utilizing the landscape‐scale variation in moisture and experimental moisture manipulations in tandem. This is similar in design to the work of Hautier and colleagues (Hautier, Niklaus, & Hector, 2009), where they accounted for the concomitant decrease in light with added nitrogen by factorially adding supplemental light to the experimental units. (For other examples, see Reich et al., 2001; Cowles et al., 2016). By doing so, studies of this design are able to increase understanding of the underlying drivers of observed changes when multiple factors change simultaneously.

In contrast to the negative effects on productivity we observed, increased temperature, as occurs in open‐top chambers, is generally expected to directly, positively affect plant growth (Rustad et al., 2001) by shifting temperature toward the photosynthetic thermal optima of the plants (Way & Oren, 2010). However, our study joins previous studies in showing negative effects of warming on plant communities, particularly in cool dryland systems (Wertin et al., 2015; Yang, Wu et al., 2011), where plants may have adapted to lower thermal optima. Thus, OTCs might create above optimal temperatures in our study system, all the while decreasing soil moisture where water is at a premium (Amthor, 2000; Bernacchi, 2002), as is backed by our observations of increased soil temperature and decreased soil moisture in the OTC treatments. Physiologically optimal temperatures are known to vary with local temperature regimes (Berry & Bjorkman, 1980; Hikosaka, 2005; Medek, Evans, Schortemeyer, & Ball, 2011), thus explaining why some ecosystems may respond more positively or negatively to changing temperatures.

Our finding of a more extreme effect of the OTCs on diversity at the upper slope location highlights landscape‐scale variation in response to climate change, as these impacts were not explained by our measured covariates, moisture and temperature. These effects could be related to differences in biophysical properties of the soil (Elmendorf et al., 2015) or to differences in species composition between the two locations. Some studies point to space‐for‐time substitution, that is, the study of spatial variation such as ecosystems existing across a gradient in field age or up a mountainside, as insight into the potential impact of the same variations over time, such as succession or global changes, as a helpful approach to understanding impacts of future climate change (Blois, Williams, & Fitzpatrick, 2013), but see (Metz & Tielbörger, 2016). If space‐for‐time is applicable in our system, then we would expect the warmer, drier upper slope to support a plant community predictive of future, climate‐induced changes on the lower slope. Conversely, watering the upper slope should make it more similar to the lower slope. Here, in contrast, the OTC alone had little consequence for plant productivity and diversity on the lower slope and watering without the OTC had little consequence on diversity for the upper slope. Moreover, the differences in productivity and diversity between slopes were much greater than those induced by any of our manipulations. Elmendorf et al. (2015) compared types of global warming studies and found space‐for‐time studies to have similar but exaggerated effects relative to experimental manipulations and interannual variation in temperature within a single site. The larger effect size in the space‐for‐time studies may be related to longer‐term changes in species composition with climatic differences or other environmental differences among sites (Elmendorf et al., 2015). It is possible that our 4‐year experiment was not long enough to induce changes consistent with large, significant pre‐existing differences between slopes.

Strong, intrinsic differences between the sites, rather than responses to different treatments, drive the changes in the correlations between our response variables (diversity and biomass) and our covariates (moisture and temperature). The correlations change sign from the cross‐slope experiment the upper slope experiment. The positive relationship between biomass and soil moisture found in the OTC × slope experiment indicates the intrinsically larger biomass and greater moisture in the lower slope location relative to the upper slope location. The impacts of treatments within a slope location were negative, more likely reflecting greater water uptake in plots with greater biomass. We were unable to untangle the causality between abiotic variables (temperature and moisture) and plant responses and so these relationships can only be discussed in terms of their correlations. There is the potential for bidirectional effects; while abiotic processes can affect plant growth and cover, plant growth and cover can also impact soil temperature and moisture via shading and soil moisture via transpiration.

For both the OTC and watering treatments, our measured environmental variables do explain much of, but not the entirety of, treatment effects on plant response variables. This is unsurprising, as both manipulations may affect environmental conditions other than soil temperature, moisture, and nitrogen. OTCs also affect air temperature, temperature extremes (Bokhorst et al., 2013), wind dynamics (Marion et al., 1997), and soil drying rates (Liancourt et al., 2012). As it has been suggested that changes in soil drying rates may be a strong driver of experimental effects of OTCs on plant productivity and diversity (Marion et al., 1997), we conducted a supplemental analysis using the variability metric of Knapp and colleagues (Knapp et al., 2002). We calculated the mean difference between soil moisture on subsequent days across two periods in June (10 days) and July (6 days) and examined correlations between this mean difference between days and our plant response variables. While Knapp et al. (2002) found variability in soil water content to be a major predictor of diversity and carbon cycling, this variability metric explained a maximum of 4% of the variability in any of our response variables. Thus, we conclude that the effect of the OTC treatment is largely via the impacts on temperature and moisture that would be expected in climate change scenarios, rather than a change in soil drying rates due to the sheltering impacts of the OTCs. We suggest further work examining impacts on other environmental factors, plant–plant interactions (Liancourt et al., 2013) and resultant ecosystem properties to understand the ecological and biophysical implications of OTCs. Such is necessary to determine how our climate manipulations align with projected global climate change and thus the applicability of global change experiments to real‐world scenarios.

Our study assists in filling a crucial gap in the literature by integrating natural landscape‐scale variation in environmental conditions with factorial interactive experimental manipulations (Sundqvist, Sanders, & Wardle, 2013). It is an important step in our understanding how global change will affect ecosystems across multiple scales and locations (De Boeck et al., 2015). More work is clearly necessary to fully understand why results of global change experiments can be system or location specific, what global change drivers may have the greatest impact on a system, and how we can manage lands to minimize negative impacts of climate change. Future studies exploring climate‐induced changes in plant community composition by focusing on responses of component species (Liu et al., 2017) in light of plant functional traits (Liancourt et al., 2015) should provide insight into the context dependency of the impacts of climate change. Understanding how temperature and soil moisture may interactively impact plant productivity, biodiversity and community composition will only grow increasingly important in the years to come as climate change continues to alter both temperature and moisture regimes.

## CONFLICT OF INTEREST

None declared.

## AUTHOR CONTRIBUTIONS

BC, PP, and BB designed the field experiment. BC, PP, BB, and PL contributed to field work. JC analyzed data and wrote the manuscript with feedback from BC and PP. All coauthors contributed feedback to the manuscript.
