# Supplementary figures and images for: Effects of increased temperature on plant communities depend on landscape location and precipitation
Source: Ecol Evol. 2018 May 8;8(11):5267–78. doi: 10.1002/ece3.3995 (PMC6010887; doi:10.1002/ece3.3995)

All hours

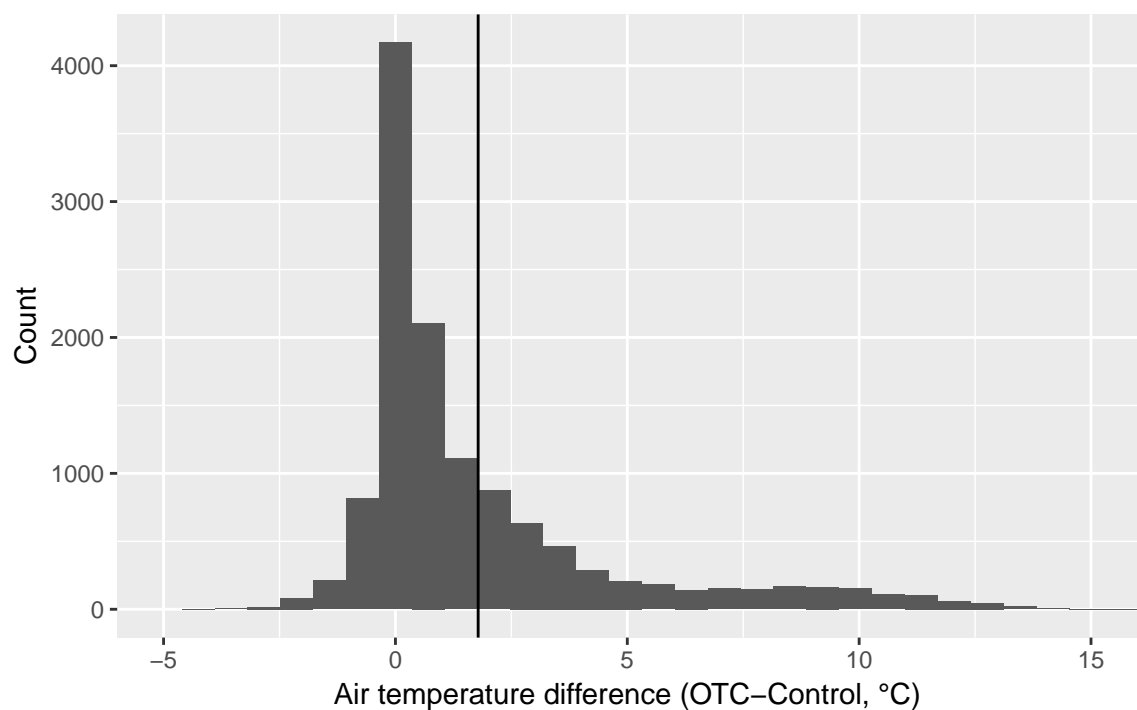

Day (10 am – 5 pm)

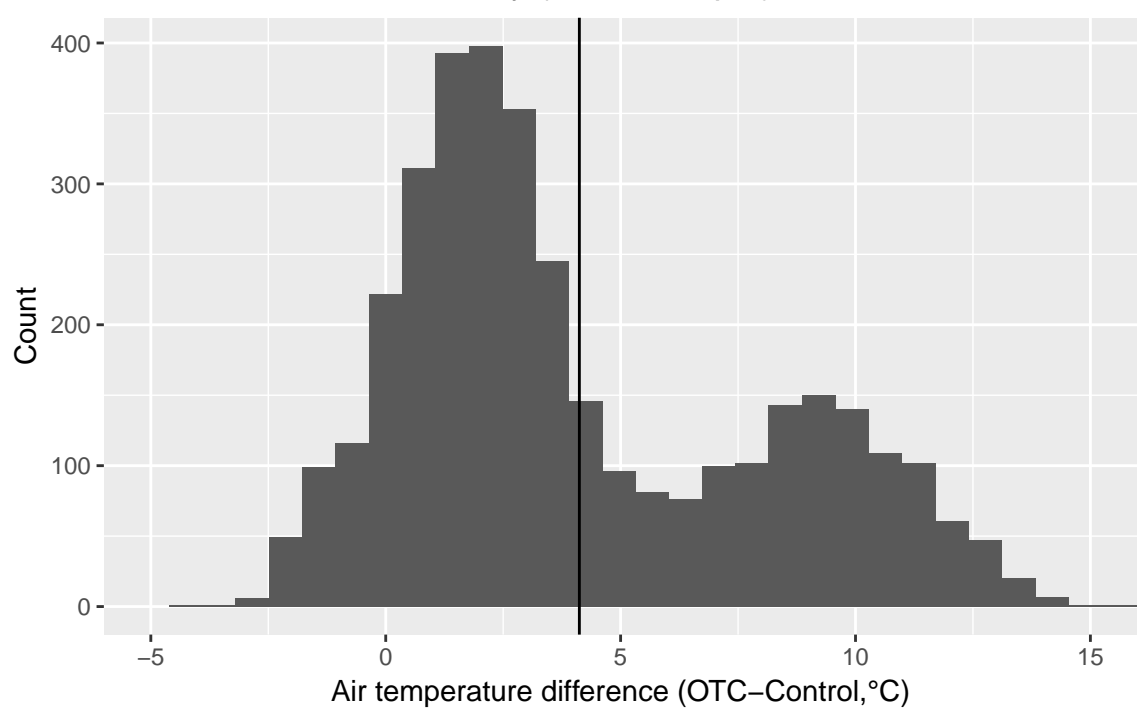

Night (10 pm – 5 am)

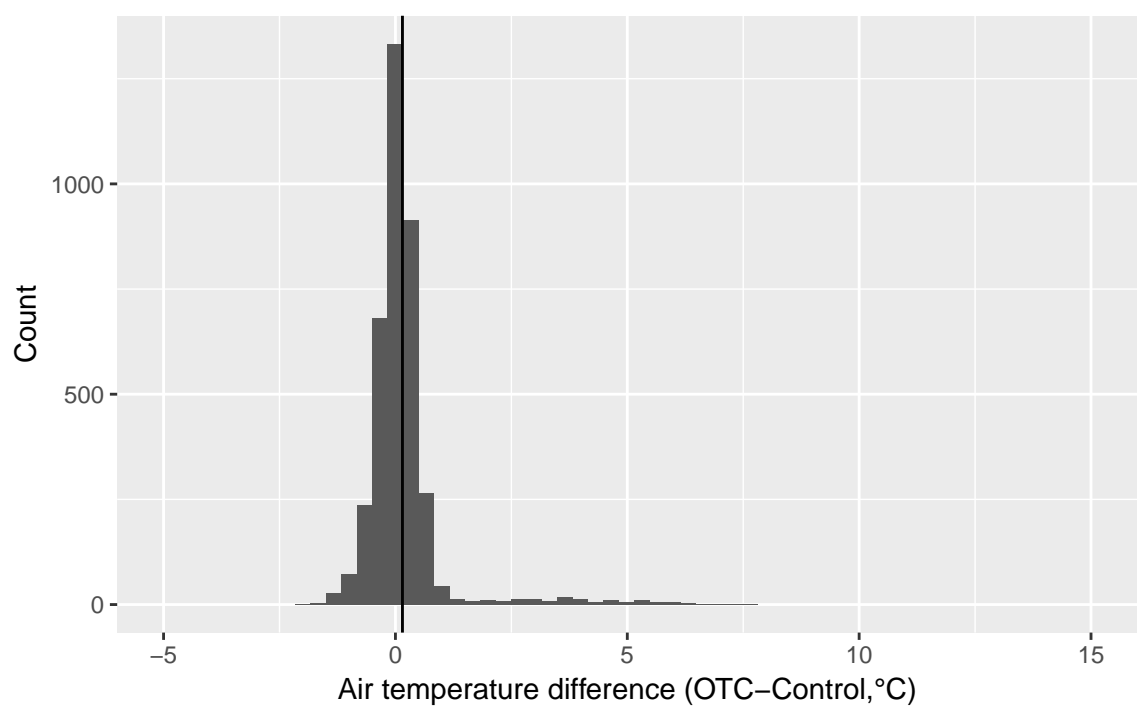

Supplement: Supplementary file 1 [file ECE3-8-5267-s001.pdf]
